# Disruption of oligodendrocyte progenitor cells is an early sign of pathology in the triple transgenic mouse model of Alzheimer's disease

**DOI:** 10.1016/j.neurobiolaging.2020.05.016

**Published:** 2020-10

**Authors:** Ilaria Vanzulli, Maria Papanikolaou, Irene Chacon De-La-Rocha, Francesca Pieropan, Andrea D. Rivera, Diego Gomez-Nicola, Alexei Verkhratsky, José Julio Rodríguez, Arthur M. Butt

**Affiliations:** a Institute of Biomedical and Biomolecular Sciences, School of Pharmacy and Biomedical Sciences, University of Portsmouth, Portsmouth, UK; b BioCruces Health Research Institute, Barakaldo, Spain; c Department of Neuroscience, University of the Basque Country UPV/EHU, Leioa, Spain; d IKERBASQUE, Basque Foundation for Science, Medical School, Bilbao, Spain; e Faculty of Biology, Medicine and Health, University of Manchester, Manchester, UK; f Achúcarro Basque Center for Neuroscience, IKERBASQUE, Basque Foundation for Science, Bilbao, Spain; g School of Biological Sciences, University of Southampton, Southampton, UK

**Keywords:** Oligodendrocyte progenitor cell, OPC, Glia, Hippocampus, Astrocyte, Amyloid β, Alzheimer's disease

## Abstract

There is increasing evidence that myelin disruption is related to cognitive decline in Alzheimer's disease (AD). In the CNS, myelin is produced by oligodendrocytes, which are generated throughout life by adult oligodendrocyte progenitor cells (OPCs), also known as NG2-glia. To address whether alterations in myelination are related to age-dependent changes in OPCs, we analyzed NG2 and myelin basic protein (MBP) immunolabelling in the hippocampus of 3×Tg-AD mice at 6 and 24 months of age, compared with non-Tg age-matched controls. There was an age-related decrease in MBP immunostaining and OPC density, together with a decline in the number of OPC sister cells, a measure of OPC replication. Notably, the loss of myelin and OPC sister cells occurred earlier at 6 months in 3xTg-AD, suggesting accelerated aging, although there was not a concomitant decline in OPC numbers at this age, suggesting the observed changes in myelin were not a consequence of replicative exhaustion, but possibly of OPC disruption or senescence. In line with this, a key finding is that compared to age-match controls, OPC displayed marked morphological atrophy at 6 months in 3xTg-AD followed by morphological hypertrophy at 24 months, as deduced from significant changes in total cell surface area, total cell volume, somata volume and branching of main processes. Moreover, we show that hypertrophic OPCs surround and infiltrate amyloid-β (Aβ) plaques, a key pathological hallmark of AD. The results indicate that OPCs undergo complex age-related remodeling in the hippocampus of the 3xTg-AD mouse model. We conclude that OPC disruption is an early pathological sign in AD and is a potential factor in accelerated myelin loss and cognitive decline.

## Highlights

• Life-long generation of myelin is the function of adult oligodendrocyte progenitor cells (OPCs).
• Age-related loss of myelin is accelerated in the 3xTg-AD mouse model of Alzheimer's disease (AD).
• OPCs are disrupted at an early stage of 3xTg-AD.
• Dysregulation of OPC and myelin loss are important biomarkers for AD-like pathology.

## Graphical abstract

**Figure:**
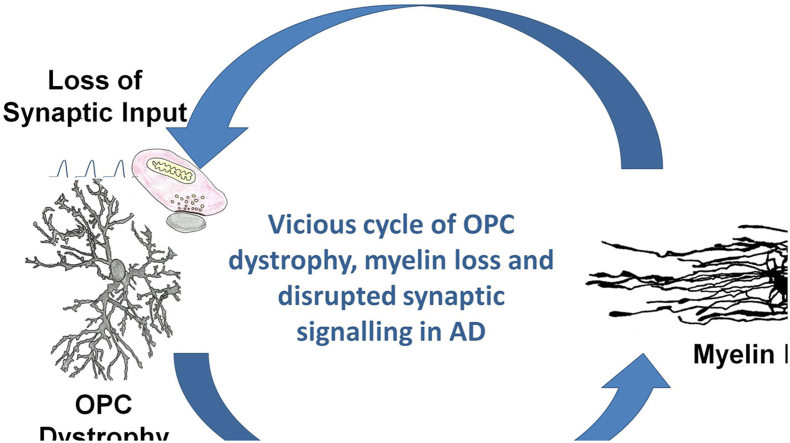

## 1 Introduction

Alzheimer's disease (AD) is a neurodegenerative disease characterised by age-related decline in learning, memory and cognition. Neuropathological hallmarks of AD are amyloid β (Aβ) plaques and neurofibrillary tangles (NFTs). In addition, myelin disruption is prominent in AD (Bartzokis, 2011), while myelin loss may predict AD onset and neurodegenerative changes in humans (Brickman et al., 2015), as well as in animal models of AD (Desai et al., 2009). Thus, disruption of myelination is a feature of AD, although the underlying causes are unresolved.

In the CNS, myelin is produced by oligodendrocytes, which are generated from oligodendrocyte progenitor cells (OPCs) (Rivers et al., 2008; Zhu et al., 2011), also known as NG2-glia or synantocytes (Butt et al., 2005). OPCs are identified by their expression of the membrane proteoglycan NG2 (Cspg4) (Stallcup, 1981), and are the main proliferative cells in the brain (Dawson et al., 2003). In the adult brain, OPCs slowly divide asymmetrically to form daughter cells that are responsible for OPC self-renewal and for generating new oligodendrocytes for myelin repair and for myelination of new connections important in learning (Hughes et al., 2018; McKenzie et al., 2014; Xiao et al., 2016; Young et al., 2013). In addition, OPCs readily respond to CNS pathology by a characteristic morphological remodeling and increased NG2 expression (Levine, 2016; Rodríguez et al., 2016). Notably, OPCs display increased senescence and disruption in the aging brain (Neumann et al., 2019; Segel et al., 2019; Sim et al., 2002), and this may be aggravated in human and mouse AD (Dong et al., 2018; Li et al., 2013; Nielsen et al., 2013; Zhang et al., 2019). These studies suggest that age-related changes in OPCs are related to a reduction in myelination in AD (Butt et al., 2019).

The triple transgenic mouse model of AD (3xTg-AD) harbors presenilin 1 (PS1; M146 V), amyloid precursor protein (APP; Swe), and tau (P301 L) transgenes, and displays progressive AD-like pathology and cognitive impairments in an age-related manner (Belfiore et al., 2019; Oddo et al., 2003; Olabarria et al., 2010). Immunohistochemical, electron microscope and imaging studies have shown myelin disruption in the hippocampus of 3×Tg-AD mice in 6 month-old mice (Desai et al., 2009, 2010; Nie et al., 2019), concomitant with the appearance of Aβ plaques (Oddo et al., 2003). Here, we have performed a detailed examination of age-related changes in OPC in the hippocampus of 3xTg-AD mouse. Morphological analysis (Olabarria et al., 2010) of total cell surface area, total cell volume, somata volume and branching of main processes demonstrated that OPC display a marked morphological atrophy at 6 months in 3xTg-AD. Morphological atrophy was collateral to a decline in the numerical density of OPC daughter cells and decreased myelin immunostaining in the hippocampus. In contrast, at 24 months in 3xTg-AD OPCs undergo a marked morphological hypertrophy and cluster around Aβ plaques with astrocytes. This study identifies complex changes in OPC morphology that are related to the pathogenesis of AD.

## 2 Materials and methods

### 2.1 Animals and tissues

All animal procedures were carried out in accordance with the United Kingdom Animals (Scientific Procedures) Act of 1986 under licence from the Home Office. All efforts were made to reduce the number of animals by following the 3 Rs. The procedure for generating 3xTg-AD mice has been described previously (Oddo et al., 2003; Rodríguez et al., 2008). The 3xTg-AD mouse line harbors the APP Swedish mutations K670 N/M671 L, the presenilin-1 M146 V mutation and the tau P301 L mutation in a C57BL6 mice background, and all 3xTg-AD and non-Tg control mice were obtained by crossing homozygous breeders. The animals were housed in same-sex cages, kept in 12 hours light-dark cycles with free access to food and water independent of the diet. at these ages that the amyloid and tau pathologies emerge resembling the human Alzheimer's disease progression. Mice aged 6 and 24 months were used, because 3xTg-AD mice have been shown to exhibit myelin disruption at 6 months prior to deposition of extracellular Aβ plaques, which are prominent at 24 months of age (Desai et al., 2010; Olabarria et al., 2010). Male 3xTgAD and non-transgenic control mice were anesthetized with intra-peritoneal injection of sodium pentobarbital (50 mg kg) at 6 and 24 months of age. The mice were perfused through the aortic arch with 3.75% acrolein (TAAB, Berkshire, UK) in a solution of 2% paraformaldehyde (Sigma, Cambridge, UK) and 0.1 M phosphate buffer (PB) pH 7.4, followed by 2% paraformaldehyde.

### 2.2 Immunohistochemistry

Brains were removed and cut into 4- to 5-mm coronal slabs of tissue containing the entire rostrocaudal extent of the hippocampus. Brain sections were postfixed in 2% paraformaldehyde for 24 hours and kept in 0.1 M PB, pH 7.4. Coronal sections of the brain were cut into 40 μm thickness using a vibrating microtome (VT1000S; Leica, Milton Keynes, UK). Free floating brain sections in 0.1 M PB, pH 7.4, were collected and stored in cryoprotectant solution containing 25% sucrose and 3.5% glycerol in 0.05 M PB at pH 7.4. Coronal sections at levels −1.58 ⁄ −2.46 mm (hippocampus) posterior to the Bregma were selected for immunohistochemistry according to the mouse brain atlas. The sections were incubated for 30 minutes in 30% methanol in 0.1 M PB and 3% hydrogen peroxide (H_2_O_2_) (Sigma). Sections were then rinsed with 0.1 M PB for 5 minutes and placed in 1% sodium borohydride (Sigma) for 30 minutes. The sections were then washed with PB profusely before rinsing in 0.1 M Trizma base saline (TS) for 10 minutes. Brain sections were then incubated in 0.5% bovine serum albumin (BSA) (Sigma) in 0.1 M TS and 0.25% Triton (Sigma) for 30 minutes. Sections were incubated for 24 hours at room temperature in primary antibody: rabbit anti-NG2, 1:400 (Millipore); rat anti-MBP, 1:400 (Millipore); mouse anti-Aβ, 1:1000 (Covance). Tissues were then washed 3 times again in TS and incubated with the appropriate secondary antibody (AlexaFluor 488, AlexaFluor 568, 1:400, Life Technologies) diluted in blocking solution for 1 hour at room temperature on an orbital shaker and protected from the light. Following secondary antibody incubation, tissues were washed 2 times with TS and 3 times with PB before being covered with mounting medium and glass coverslips ready for imaging.

### 2.3 Confocal microscopy and image analysis

Images were captured using a Zeiss Axiovert LSM 710 VIS40S confocal microscope and maintaining the acquisition parameters constant to allow comparison between samples within the same experiment. Acquisition of images was done with x20 objective for cell counts and analysis of MBP immunostaining; x40 objective was used to examine relationships between OPCs and Aβ plaques; x100 objective was used for OPC 3D reconstruction and morphological analysis, with *z*-stacks formed by 80–100 single plains with an interval of 0.3 μm.

### 2.4 Quantification

Cell counts for OPCs were performed in a constant field of view (FOV) of 100 μm x100 μm in projected images of z-stacks of 10 optic sections with 1 μm interval. Morphological analyses of NG2-glia was performed as previously used for astrocytes in the 3xTg-AD mouse (Olabarria et al., 2010), using the *Cell Analyst* program and morphological calculations detailed in (Chvátal et al. 2007). In brief, the *Cell Analyst* program builds a detailed 3-D reconstruction of the cell based on a series of high resolution confocal z-stacks 0.3 μm apart, which defines the precision of the analysis (Chvátal et al., 2007); we used 5 digital filters (average 3 × 3, convolution, gauss 5 × 5, despeckle, simple objects removal) and a threshold of 50 to determine the surface and volume of the NG2-positive OPCs (Olabarria et al., 2010). Relative density of MBP immunostaining was measured from a constant field of view using ImageJ; a threshold was set from negative controls and parameters were kept constant to avoid experimental errors and/or bias. Results are expressed as mean ± SEM and statistical differences were determined by one-way ANOVA and Newman–Keuls multiple comparison *post-hoc* analysis or unpaired t-tests, as appropriate, using Graphpad Prism5.0.

## 3 Results

### 3.1 Age-related changes in OPC numbers and MBP immunolabelling

**Fig. 1:**
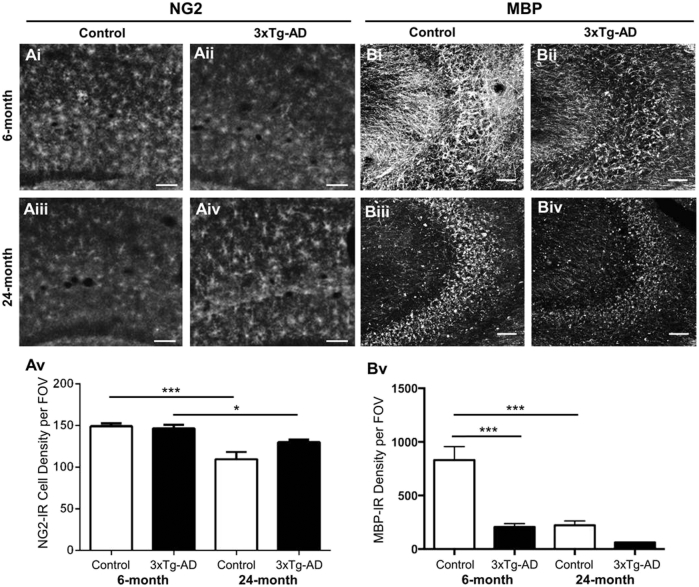
Age-dependent changes in OPCs and myelin in the hippocampus of 3xTg-AD and age-matched non-Tg controls. (Ai-iv, B-iv) Confocal images illustrating the hippocampus immunolabelled with the OPC marker NG2 (Ai-iv) and the myelin marker MBP (Bi-iv). Scale bars = 50 μm. (Av, Bv) Bar graphs showing the numerical density of NG2+ OPCs (Av) and MBP immunoreactive intensity (Bv) in the hippocampus; data are mean ± SEM, ∗*p* < 0.05, ∗∗*p* < 0.01, unpaired t-tests.

Disruption of oligodendrocytes and myelin is an early event in the hippocampus of 3xTg-AD mice (Desai et al., 2009, 2010), but it is unclear how this is related to age-related changes in OPCs, which are responsible for life-long generation of myelinating oligodendrocytes (Young et al., 2013). To examine this, we performed immunostaining for NG2 and MBP in 6 and 24 month old 3xTg-AD, compared to age-matched controls (Fig. 1). NG2-positive OPCs were widely distributed throughout the hippocampus of both 3xTg-AD and non-Tg mice (Fig. 1Ai-iv), and cell counts indicated there was a decrease in the overall numerical density of OPCs between 6 months and 24 months in both 3xTg-Ad and non-Tg controls (Fig. 1Av, unpaired t-tests). In addition, we observed an age-related decrease in MBP immunostaining between 6 months and 24 months in non-Tg controls (Fig 1Bi, iii), however this decrease occurred earlier in 3xTg-AD at 6 months (Fig. 1Biii), without further change at 24 months (Fig. 1Biv). Quantitative analysis confirmed that MBP immunofluorescence intensity was significantly less in 6-month 3xTg-AD hippocampus compared to age-matched non-Tg controls (Fig. 1Bv; *p* < 0.001) and equivalent to the level seen in 24-month controls. The results demonstrate a decline in OPCs and MBP with physiological aging, but although OPC numerical density was unaltered in 3xTg-AD, the loss of MBP was significantly accelerated.

### 3.2 Age-related changes in OPC sister cells

**Fig. 2:**
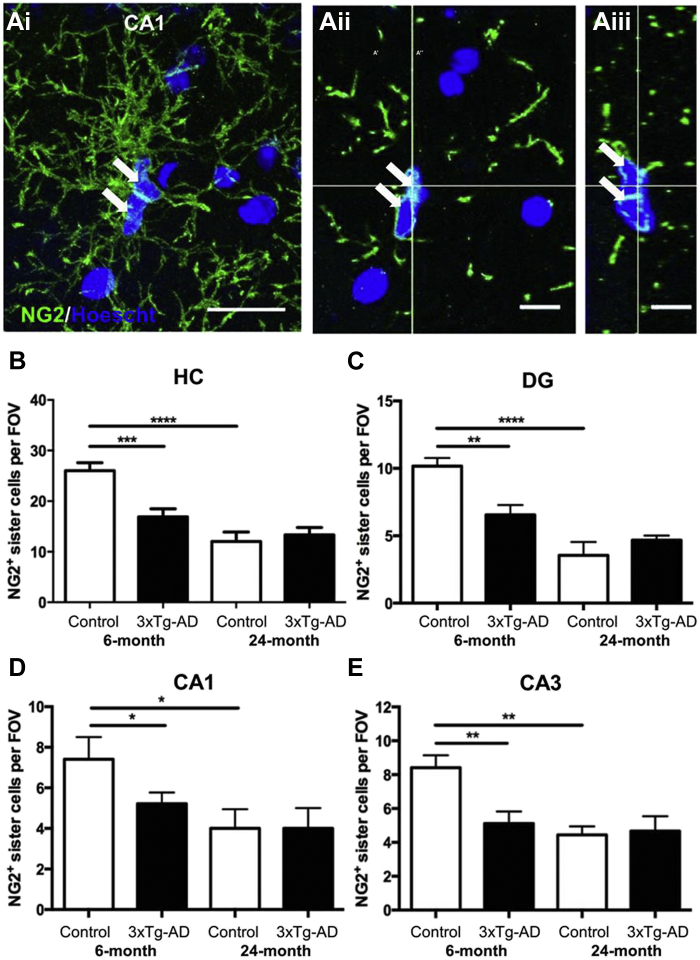
Age-dependent changes in OPC daughter cells in hippocampus of 3xTg-AD and age-matched non-Tg controls. (A) Confocal images illustrating OPC duplets (arrows), in the CA1 area of the hippocampus of 3xTg-AD mice immunolabelled for NG2 (green) and counterstained with the nuclear marker Hoechst blue (blue). Maximum intensity *z*-stack projection (Ai), together with single z-section (Aii) and orthogonal section through the *x-x* plane (Aiii), showing juxtaposed duplets of recently divided OPC daughter cells. Scale bars = 20 μm in Ai and 10 μm in Aii, Aiii. (B–E) Quantification of sister cells per constant field of view (FOV) in the hippocampus overall (B), the dentate gyrus (DG) (C), CA1 (D) and CA3 (E); data are mean ± SEM from 3 sections per animal, *n =* 3 animals. *∗p* < 0.05, *∗∗p* < 0.01, *∗∗∗p* < 0.001, unpaired t-tests. (For interpretation of the references to color in this figure legend, the reader is referred to the Web version of this article.)

Similarly to stem cells, OPCs self-maintain by undergoing asymmetric division and recently divided cells occur as closely associated duplets or triplets of ‘daughter cells’ that either self-sustain OPCs or proceed to differentiate into oligodendrocytes (Boda et al., 2014). Hence, the frequency of OPC duplets/triplets is a measure of cell division and OPC regenerative potential (Boda et al., 2014). To examine this in aging, NG2 immunostaining was performed together with nuclear labeling with Hoechst blue to highlight groups of 2 or more juxtaposed OPC daughter cells (Fig. 2). OPC doublets and triplets were prominent throughout the hippocampus in 6 months non-Tg controls, exemplified in the CA1 (Fig. 2A). Quantitation shows a significant decrease in OPC daughter cells between 6 and 24 months in non-Tg controls in the hippocampus overall (Fig. 2B; *p* < 0.001, unpaired t-tests), and regional analyses demonstrated equivalent decline in the dentate gyrus (DG), and CA1 and CA3 regions (Fig. 2C–E). At 6 months, the number of OPC daughter cells was significantly less in 3xTg-AD hippocampus than age-matched controls (Fig. 2B; *p* < 0.001), and declined to the level seen at 24-months in controls (Fig. 2B–E). There was no further change in OPC daughter cells at 24 months in 3xTg-AD, which was equivalent to age-matched controls (Fig. 2B–E). The results support a decline in OPC replication in physiological aging that is observed at a younger age in 3xTg-AD.

### 3.3 Age-related changes in OPC morphology

**Fig. 3:**
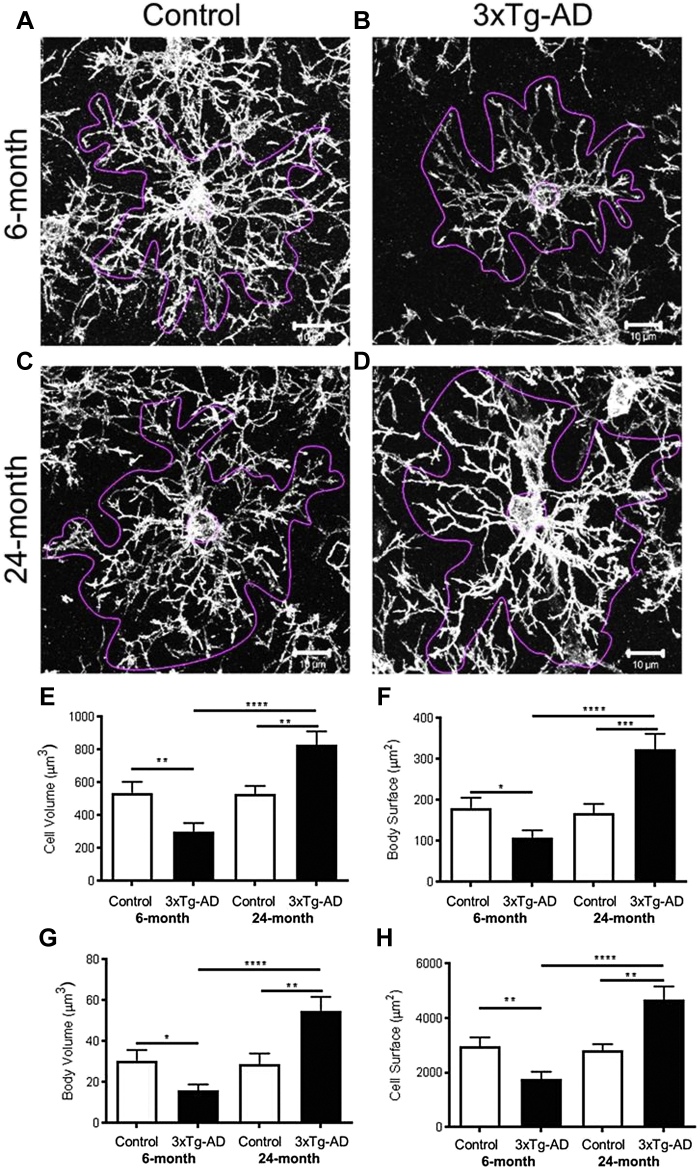
Age-dependent changes in OPC morphology in the CA1 hippocampus of 3xTg-AD and age-matched non-Tg controls. (A–D) Representative confocal images of NG2 immunopositive OPCs in the CA1 region of the hippocampus, illustrating in magenta the outline used for analysis of individual cells using the *Cell Analyst* program (Chvátal et al., 2007); scale bars = 10 μm. (E–H) Quantification of OPC total cell volume (E), cell body surface area (F), cell body volume (G) and total cell surface area (H); data are mean ± SEM from 25 cells from 3 sections per mouse, where *n =* 3 or 4). ∗*p* < 0.05, ∗∗*p* < 0.01, ∗∗∗∗*p* < 0.0001 unpaired t-tests.

Changes in the cellular morphology of OPCs is a characteristic of the response to CNS pathology (Levine, 2016, Rodríguez et al., 2016) and has been reported in human AD and mouse models of the disease (Dong et al., 2018, Li et al., 2013, Nielsen et al., 2013, Zhang et al., 2019). To examine whether this occurs in 3xTg-AD, we performed a detailed morphological analysis of NG2 immunostained OPCs in the hippocampus at 6 and 24 months (Fig. 3), using the *Cell Analyst* program and morphological parameters detailed by (Chvátal et al. 2007), as described previously for astrocytes in the 3xTg-AD model (Rodríguez et al., 2013). The results show no significant difference in the morphology of OPCs at 6 and 24 months in non-Tg controls (Fig. 3A and C), also confirmed by quantification of multiple cellular morphological parameters (Fig. 3E–H). In contrast, at 6 months OPCs displayed marked morphological atrophy in 3xTg-AD, compared to controls (Fig. 3A and B), exhibiting a significant decrease in overall cell size and size of cell somata (Fig. 3E–H). Conversely, at 24 months OPCs displayed marked hypertrophy in 3xTg-AD, compared to age-matched controls (Fig. 3C and D), and 6 months 3xTg-AD (Fig. 3B), with significantly larger cell volume and surface area (Fig. 3E and H) and of cell somata volume and surface area (Fig. 3F and G). It is notable that OPC have circular domains in the *x-y* plane, but are extremely flat in the *z*-plane, and their somata are not spherical, but instead appear as a ‘fjord-like’ in the *x-y* planes, and extremely thin in the *z*-plane (Fig. 2Aii, Aiii), hence surface-volume relationships are not simple cubic as for a sphere. Overall, the surface-volume ratio is ≥10 in OPC, as it is in astrocytes, although OPC are much smaller and the overall values are correspondingly 2–3 times smaller than equivalent measurements in astrocytes (Chvátal et al., 2007; Olabarria et al., 2010). The results demonstrate OPCs go through complex morphological changes in 3xTg-AD that are not observed in natural aging, characterised by shrinkage in early AD, followed by a clear hypertrophy at late stages, indicative of a reactive metamorphosis.

### 3.4 OPCs are closely localised with Aβ plaques in 3xTg-AD

**Fig. 4:**
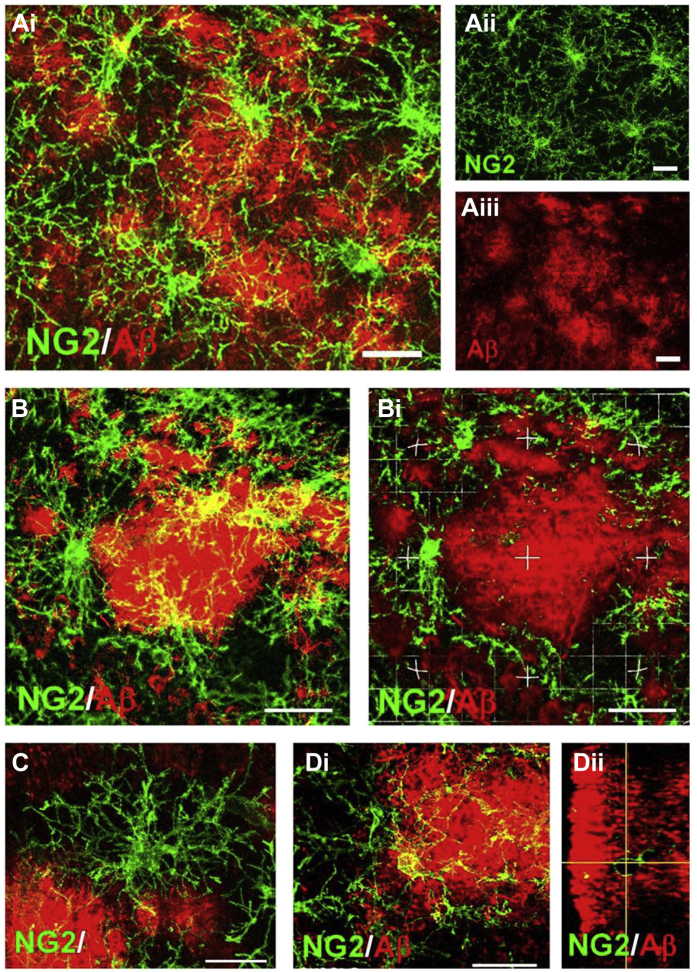
Relationships between OPCs and Aβ plaques in the hippocampus of 24-month 3xTg-AD. Confocal images of coronal brain sections from 24-month 3xTg-AD double immunostained for NG2 (green) and Aβ (red). (A) Overview of the association of OPCs with Aβ in the CA1 area of the hippocampus, with merged image (Ai) and indvidual channels for NG2 (Aii) and Aβ (Aiii). (B) OPCs clustering around Aβ plaque in maximum intensity *z*-stack projection (Bi) and single z-section (Bii). (C) Maximum intensity *z*-stack projection of OPC within the boundary of multiple Aβ plaques. (D) OPC embedded within an Aβ plaque in maximum intensity *z*-stack projection (Di) and orthogonal section (Dii). Scale bars = 50 μm in A, 25 μm in B and 20 μm in C, D. (For interpretation of the references to color in this figure legend, the reader is referred to the Web version of this article.)

**Fig. 5:**
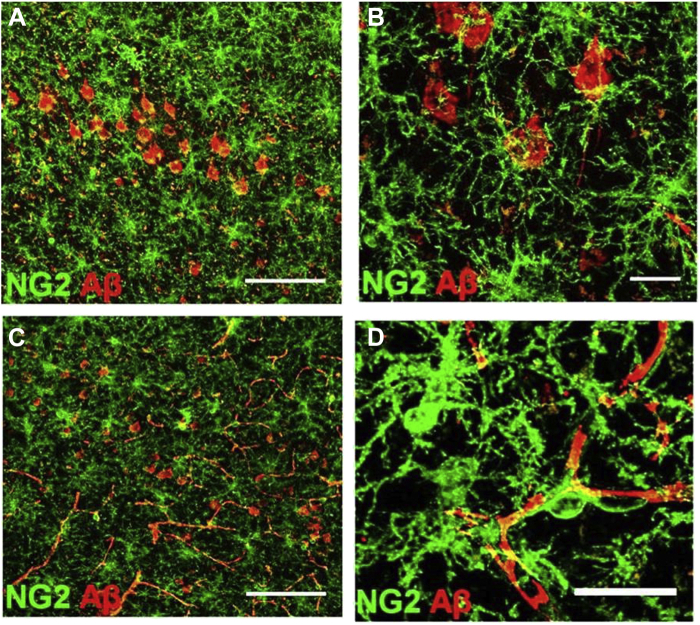
Relationship of OPCs with intraneuronal and vascular Aβ in 3xTg-AD. Confocal images of 24 months 3xTg-AD cortex double immunostained for NG2 (green) and Aβ (red). OPCs are directly apposed to Aβ containing neurones (A, B) and blood vessels (C, D). Scale bars = 100 μm in A, C, and 20 μm in B, D. (For interpretation of the references to color in this figure legend, the reader is referred to the Web version of this article.)

Neuropathological changes and Aβ plaque load become significantly greater with age in 3xTg-AD mice in AD-relevant brain regions, including the hippocampus (Belfiore et al., 2019, Oddo et al., 2003). There is evidence from in vitro studies that Aβ is toxic for OPCs (Desai et al., 2011) and induces morphological condensation (Nielsen et al., 2013). However, we did not observe a decrease in OPC in 3xTg-AD and cells were hypertrophic at 24 months. We therefore performed double immunfluorescence labeling to examine the relations between OPCs and Aβ plaques in 24 months 3xTg-AD (Fig. 4). The results demonstrate that hypertrophic OPCs are adjacent to Aβ plaques throughout the hippocampus (Fig. 4A). Higher magnification imaging in the CA1 hippocampal area demonstrates the intimate relationships between OPCs and Aβ plaques (Fig. 4B–D). Large Aβ plaques are circumscribed by OPCs and infiltrated by their processes (Fig. 4B). OPCs were observed to be located both between multiple plaques that they contact (Fig. 4C), as well as being embedded within Aβ plaques (Fig. 4D). In the cortex, intraneuronal and vascular Aβ immunoreactivity was prominent in 24 months 3xTg-AD (Fig. 5A and C), and OPCs were directly apposed to these, surrounding them with their processes (Fig. 5B and D). The results demonstrate that OPCs are intimately associated with Aβ plaques in 3xTg-AD, consistent with a recent study in human AD (Zhang et al., 2019).

### 3.5 Interrelations between OPCs and astrocytes in Aβ plaques

**Fig. 6:**
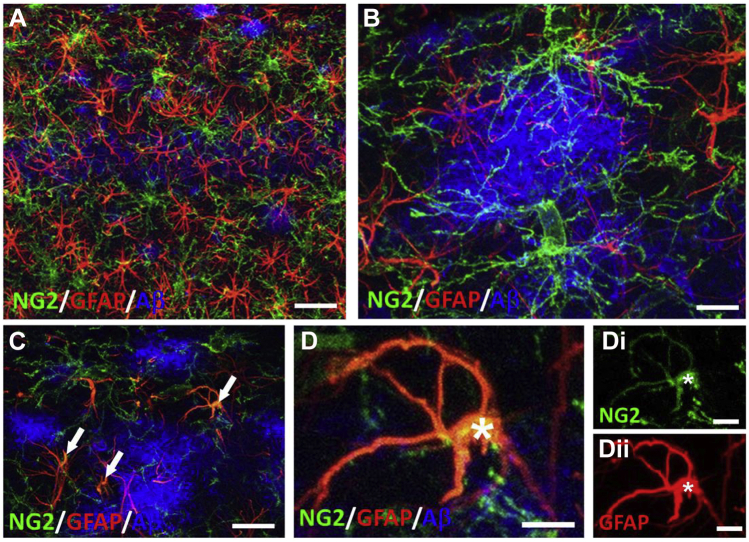
Interrelations between OPCs and astrocytes with Aβ plaques in the 24-month 3xTg-AD hippocampus. Confocal images of CA1 region triple immunostained for NG2 (green), GFAP (red) and Aβ (blue). (A) OPCs and astrocytes are uniformly distributed in the CA1 layer with overlapping process domains. (B) OPCs and astrocytes are associated with the same Aβ plaques, with processes intertwined within Aβ plaques. (C) Large Aβ plaque containing cells co-expressing NG2 and GFAP (arrows, colocalization appears yellow). (D) High magnification maximum intensity *z*-stack projection of an NG2+/GFAP+ cell (asterisk), illustrating merged image (D), together with individual channels for NG2 (Di) and GFAP (Dii). Scale bars = 50 μm in A, 10 μm in B, D, and 30 μm in C. (For interpretation of the references to color in this figure legend, the reader is referred to the Web version of this article.)

In 3xTg-AD, astrocyte hypertrophy is associated with Aβ plaques (Olabarria et al., 2010), and there is evidence astrocytes play a role in the clearance and removal of Aβ (Nielsen et al., 2010). The close associations of OPC with Aβ plaques demonstrated above stimulated us to examine their interrelationships with astrocytes in the 24-month 3xTg-AD hippocampus (Fig. 6). Triple immunostaining for NG2, GFAP and Aβ shows that both astrocytes and OPCs are densely distributed throughout the hippocampus in association with Aβ deposits (Fig. 6A), and the processes of OPCs and astrocytes overlap extensively, contacting and infiltrating proximal Aβ plaques (Fig. 6B). Furthermore, in the hippocampus of 24 months 3xTg-AD we rarely observed cells co-immunostained for NG2 and GFAP (Fig. 6C and D). NG2 positive astrocytes have been reported in human AD in individuals with high Aβ plaque load (Nielsen et al., 2013), and comparison with NG2+/GFAP− OPCs and NG2−/GFAP+ astrocytes in the same field of view in our study indicates NG2+/GFAP+ cells had a morphology more akin to astrocytes than OPCs (Fig. 6D). Our results demonstrate that OPCs and astrocytes cluster around the same Aβ plaques in 3xTg-AD.

## 4 Discussion

Age-related loss of myelin is a feature of normal aging and has been shown to be exacerbated in human AD (Bartzokis, 2011; Brickman et al., 2015) and in multiple animal models of AD-like pathology (Desai et al., 2009; Dong et al., 2018). Here, we confirm previous observations (Desai et al., 2009, 2010), by showing that compromised myelination is evident at 6 months of age in 3xTg-AD, to a degree comparable to that observed at 24 months in natural aging. The maintenance of myelination in the adult brain is an ultimate function of OPCs, which divide and regenerate oligodendrocytes more slowly in the aging brain (Psachoulia et al., 2009; Young et al., 2013). Our data shows an age-related decrease in the number of OPC daughter cells occurred significantly earlier, at 6 months, in 3xTg-AD, suggesting OPC self-renewal is compromised at early stages of AD-like pathology. Notably, OPCs displayed marked cellular shrinkage at 6 months in 3xTg-AD. In contrast, OPCs were hypertrophic at 24 months in 3xTg-AD and, together with astrocytes, were closely associated with Aβ plaques (Li et al., 2013; Nielsen et al., 2010). Our findings indicate complex changes in OPCs are important markers for early and late stages of AD-like pathology, which may be a major factor in myelin loss.

Imaging studies in 3xTg-AD show pronounced myelin changes in the fimbria, which acts as the major output tract of the hippocampus (Nie et al., 2019), and our results support evidence that myelination is reduced in 3xTg-AD (Desai et al., 2009, 2010, 2011; Mastrangelo and Bowers, 2008). These results in 3xTg-AD mice correlate well with imaging and postmortem studies in human AD demonstrating widespread abnormalities in oligodendrocytes and myelin, including reduced MBP (Bartzokis, 2011; De Rossi et al., 2016; Ihara et al., 2010; McKenzie et al., 2017; Nasrabady et al., 2018; Roher et al., 2002). Indeed, white matter changes are an early feature of human AD (Fischer et al., 2015; Hoy et al., 2017), and the premature loss of MBP immunostaining observed in our study is consistent with myelin loss being an early event in AD pathology and cognitive decline (Butt et al., 2019; Nasrabady et al., 2018; Wang et al., 2020). Certainly, myelin disruption is coincident with the earliest signs of cognitive impairment and neuropathology in 3xTg-AD (Billings et al., 2005; Oddo et al., 2003). Furthermore, myelin disruption has been shown to result in axonal and neuronal degeneration (Stassart et al., 2018), and myelin injury and loss of oligodendrocytes in AD is associated with axon degeneration and amyloid plaques (Mitew et al., 2010; Zhan et al., 2015). The causes of myelin loss in AD are likely to include direct Aβ toxicity (Desai et al., 2011), as well as glutamate, metabolic and iron dyshomeostasis (Ndayisaba et al., 2019; Wang and Reddy, 2017; Yin et al., 2016). Our results indicate disruption of OPCs is another key event that may contribute to myelin loss in early AD.

At early stages of disease progression in 3xTg-AD, OPCs displayed a striking morphological atrophy, manifested by decreased cell surface area and volume, concomitant with a >30% decrease in OPC daughter cells, a measure of their recent cell division (Boda et al., 2014). The asymmetric division of OPCs is critical for self-replication and the life-long generation of new myelinating oligodendrocytes (Dimou et al., 2008; Kang et al., 2010; Rivers et al., 2008; Zhu et al., 2008). Hence, the early atrophy of OPCs and apparent decrease in self-renewal indicates disruption of OPCs may be a key factor in the accelerated loss of myelin in 3xTg-AD. Notably, OPCs extend processes to contact glutamatergic synapses in the hippocampus (Bergles et al., 2000), and glutamatergic signaling promotes OPC proliferation and differentiation (Chen et al., 2018; Wake et al., 2011). It is significant, therefore, that synaptic dysfunction, in particular disruption of glutamatergic signaling, is an early event in 3xTg-AD (Chakroborty et al., 2019; Clark et al., 2015; Oddo et al., 2003), and is a major component of human AD (Crimins et al., 2013). Astroglial glutamate homeostasis is also dysregulated in 3xTg-AD (Kulijewicz-Nawrot et al., 2013; Olabarria et al., 2010), which would further exacerbate glutamate signaling onto OPCs. The atrophy of OPC processes is contemporaneous with disruption of synaptic signaling, suggesting this is an important factor in the observed decline in OPC self-renewal and reduced capacity for replacing myelin lost in AD (Rivera et al., 2016). Overall, OPC numbers did not decline until later ages in 3xTg-AD, implying the accelerated decline in myelination in 3xTg-AD was not directly related to a loss of OPCs, but may reflect reduced differentiation and OPC senescence (Zhang et al., 2019). At later stages of disease progression OPCs displayed a marked hypertrophy in 3xTg-AD, with increased cell volume and surface area, thicker processes and enlarged cell bodies, characteristic of an injury response in OPCs (Ong and Levine, 1999). Hypertrophic OPCs contacted Aβ plaques together with astrocytes, which have been described previously (Rodríguez et al., 2008). These findings are consistent with OPCs being relatively resistant to Aβ (Horiuchi et al., 2012), and there is evidence OPCs are involved in Aβ clearance (Li et al., 2013; Nielsen et al., 2010).

In conclusion, the results demonstrate that OPCs undergo complex changes that are related to accelerated myelin loss in 3xTg-AD. This study highlights the importance of OPCs in AD pathogenesis.

## Disclosure statement

AB and AR are shareholders in the company GliaGenesis. The authors declare no other conflicts.

## CRediT authorship contribution statement

**Ilaria Vanzulli:** Formal analysis, Investigation, Methodology, Writing - original draft. **Maria Papanikolaou:** Formal analysis, Investigation, Methodology, Validation. **Irene Chacon De-La-Rocha:** Investigation, Methodology, Validation. **Francesca Pieropan:** Investigation, Methodology, Validation. **Andrea D. Rivera:** Investigation. **Diego Gomez-Nicola:** Writing - review & editing. **Alexei Verkhratsky:** Conceptualization, Writing - review & editing. **José Julio Rodríguez:** Conceptualization, Resources, Software, Writing - review & editing. **Arthur M. Butt:** Conceptualization, Data curation, Formal analysis, Funding acquisition, Project administration, Resources, Supervision, Validation, Visualization, Writing - original draft, Writing - review & editing.
